# Supplementary material for: Subpopulations of neurons in lOFC encode previous and current rewards at time of choice
Source: eLife. 2021 Oct 25;10:e70129. doi: 10.7554/eLife.70129 (PMC8616578; doi:10.7554/eLife.70129)
Supplement: Peer review history

# Editor report: Editor's evaluation

**DOI:** 10.7554/eLife.70129.sa0

This study used advanced statistical methods to classify the responses of neurons in the lateral orbito-frontal cortex (lOFC) of rats performing economic choices. The authors report that lOFC neurons can be classified into a small number of distinct types based on their characteristic temporal dynamics and preferences for task variables (e.g., choice, success, reward history). The results will be of interest to neuroscientists studying prefrontal cortex, as they demonstrate order amidst what would appear as disorganized diversity, and suggest that some neuronal types play specific functional roles; for instance, in integrating previous trial outcomes into a current choice.

# Decision letter

**DOI:** 10.7554/eLife.70129.sa1

**Affiliations:** Wake Forest School of Medicine United States; Wake Forest School of Medicine United States

In the interests of transparency, eLife publishes the most substantive revision requests and the accompanying author responses.

**Decision letter after peer review:**

Thank you for submitting your article "Subpopulations of neurons in lOFC encode previous and current rewards at time of choice" for consideration by *eLife*. Your article has been reviewed by 3 peer reviewers, including Emilio Salinas as the Reviewing Editor and Reviewer #1, and the evaluation has been overseen by Floris de Lange as the Senior Editor. The following individual involved in review of your submission has agreed to reveal their identity: Paul Masset (Reviewer #2).

The reviewers have discussed their reviews with one another, and the Reviewing Editor has drafted this to help you prepare a revised submission.

Essential revisions:

The reviewers agreed that this is a valuable and interesting study (see reviews below). They suggested additional analyses that would strengthen the main conclusion of the study, because the clustering methods are not conclusive about the presence of distinct subpopulations. The thought is that any additional evidence in support of the central claims would be helpful.

1) The authors should consider separation metrics that have been used in previous studies (the silhouette score and the adjusted rand index) and compare the optimal number of clusters found with these metrics with their analysis using the gap statistics. This would give better insight into the parameters controlling the complexity of the responses at the level of populations. See comment from Rev 2.

2) It would also be interesting if the authors compared the properties of neurons in cluster 3 to those of striatum-projecting neurons (and their associated cluster) found in a previous study (Hirokawa et al., 2019). Potentially, this could show that the clustering methods presented here can robustly identify similar populations of neurons across behavioral tasks, and would also provide a potential mechanistic basis for the learning effects mediated by OFC. See comment from Rev 2.

3) If data are available/appropriate, determine whether neurons have narrow or broad spikes, thus providing another potential criterion for characterizing the clusters. See comment from Rev 3.

4) In Figure 2C it seems that clusters largely differ by their late responses at the end of the trials. Does a cluster analysis based on the late parts of the PSTHs lead to similar results to those found? See comment from Rev 3.

5) The endorsement of adaptive value coding as something that OFC is dedicated to is perhaps a bit too optimistic, considering that only 15% of neurons demonstrated it (see comment from Rev 1). The authors should consider a more balanced discussion of this point.

6) The authors mention that the neurons in cluster 3 might support the integration of reward signals, but it is largely unclear why, especially from a computational point of view. Why do history and current trial reward signals ought to be integrated in this task? Spelling this out would be useful.

*Reviewer #1:*

This is a clear and concise manuscript that aims to understand the diversity of responses observed in the lOFC, a structure implicated in the assignment of value to different available choices, and in monitoring the outcomes of those choices. The work has many technical strengths:

– Multiple statistical methods and measures are used to determine whether there are identifiable neuronal types in lOFC, and what their distinct properties are.

– The methods are comprehensive, well described, and attempt a relatively unbiased, agnostic characterization of the recorded neural activity.

– The behavioral task is rich, and the results are a natural complement to a previous paper describing other aspects of this same dataset.

– The analysis is thorough. The conclusions are judicious, and are justified by the data.

Few weaknesses were noted. Perhaps the case in favor of adaptive coding is a bit exaggerated, but this is a minor issue of interpretation.

The most notable result is that one particular group of neurons encodes past reward history just before an impending choice, and may play a unique role in guiding or biasing the choice accordingly. Although this is not hugely surprising, and further experiments would be needed to prove this idea, it does demonstrate a high degree of order and functional specialization that would not be apparent without careful classification of neuronal properties. More generally, the methods and results should resonate with a wide audience, because classifying a large population of neurons into functionally significant subgroups is a problem that systems neuroscientists face in virtually every task and neural circuit.

*Reviewer #2:*

The firing patterns of single neurons in prefrontal cortex exhibit a large functional diversity. However, recent work has shown that behind this diversity there is significant structure and that this structure could be supported by the cell types and projection targets of prefrontal neurons. In this paper, the authors refine our understanding of this structure by developing new analysis methods. This paper re-analyzes single neuron data recorded in the lateral orbitofrontal cortex (OFC) in a complex behavioral task in which rats must choose between two options of varying reward probability and reward size. The authors' goal is to use dimensionality reduction methods and clustering to identify distinct neural subpopulations that underlie computations thought to be performed in OFC.

The authors use either the peri-stimulus time histograms (PSTHs) or tuning to specific task features to show that the populations of OFC neurons cluster in distinct subpopulations. Across the two methods, two clusters share a large number of cells, and one of these appears to carry a reward history signal. Just before the choice (and therefore the outcome of the current trial), one of the clusters exhibits an increased selectivity for the outcome of the previous trial. This is the kind of signal the authors were looking for and is consistent with the role of OFC in learning. The specificity here is that this signal is confined to a subpopulation of OFC neurons identified through the clustering procedure.

A key strength of the paper is that they use several methods to show that the structure they identify is robust to the features used for clustering and that the clusters exhibit a diversity of functional tuning to behaviorally relevant task parameters (reward, choice, etc). Specifically, they show that two major clusters of cells are conserved whether the clustering is performed on the temporal dynamics of the PSTHs or the tuning to task parameters. They then performed a Generalized Linear Model (GLM) analysis to identify the time course of tuning to several behaviorally relevant task variables. Again, they used two metrics of selectivity (coefficient of partial determination and mutual information) and these two metrics give similar results, strengthening their conclusions. Across the clusters the neurons exhibit broadly similar time courses of tuning. The two most striking departures from the population average occur for neurons in the two clusters that are most conserved across clustering techniques. This suggests that the neurons in these two clusters convey specific task related information. One of these clusters shows an increase in selectivity about the outcome in the previous trial right before the outcome in the current trial is revealed. This selectivity is highlighted as a possible contribution of this specific subpopulation to the known role of OFC in learning in uncertain environments.

This is an interesting paper that goes further than previous work in the prefrontal cortex in characterizing the structure of neuronal populations by using a novel combination of analysis techniques. However, the authors could perform a few additional analyses that would strengthen the paper, allow a more direct comparison with other results in the literature and bring more biological insights. The type of combined analysis presented here (clustering using different types of features, GLM reconstruction of the firing rates, etc) is likely to become a standard prerequisite when analyzing recordings from single neurons in behaving animals.

Comments for the authors:

The authors present a nice set of analyses that are well executed but a few more characterizations of the results could strengthen the biological findings.

1. As the authors point out, their analysis identifies fewer clusters than previous work attempting to cluster the functional properties of OFC neurons. The dimensionality of representations in neural circuits is thought to be partially constrained by task complexity and it would strengthen the authors' argument if they showed that this result holds across different cluster separation metrics. The authors should use both metrics that have been used in previous studies (the silhouette score and the adjusted rand index) and compare the optimal number of clusters found with these metrics with their analysis using the gap statistics. This would give a better insight into the parameters controlling the complexity of the responses at the level of populations. This comparison would provide some evidence as to whether the dimensionality is constrained by task complexity or by the structure of the neural circuits in prefrontal cortex and strengthen the biological findings in the paper.

2. On that note, it would be interesting if the authors compared the properties of neurons in cluster 3 to those of striatum-projecting neurons (and their associated cluster) found in a previous study (Hirokawa et al., 2019). Here, the authors show that neurons in cluster 3 have a strong response to the outcome (Figure 3) and to the reward history (Figure 4). Furthermore, they have an elevated firing rate prior to the trial start (Figure 1). It would be interesting to see the PSTHs for these neurons into the next trial separated by whether the trial was rewarded or not. If these neurons follow the same pattern as the striatum-projecting neurons in the previous study, it could indicate that the clustering method presented here can robustly identify similar populations of neurons across behavioral tasks. This would also provide a potential mechanistic basis for the learning effects mediated by OFC.

*Reviewer #3:*

I think this is a well-done analysis, but I see some potential limitations in the methods and in the conclusions. First, it is unclear whether the observed clusters actually correspond to distinct neuronal types, or whether they are just functionally different. One potential analysis (if data is available) is to study whether neurons have narrow or broad spikes, thus giving additional insights as to the nature of the clusters.

In Figure 2C it looks that clusters largely differ by their late responses at the end of the trials. Does a cluster analysis based on the late parts of the PSTHs lead to similar results to those found?

The authors show that cluster 3 exhibit the most prominent response to reward, but based on the PSTH clustering, the difference is very small. In addition, the increase in CPD for encoding reward history (Figure 5) is very small, although real. In principle I don't have any problems with the small effect sizes but, given that the authors make somehow strong claims about that, I am worried about the implications of the observation. The authors claim that this might support integration of reward signals, but it is largely unclear why, especially from a computational point of view: why do history and current trial reward signals ought to be integrated in this task?

# Reply: Author response

**DOI:** 10.7554/eLife.70129.sa2

Essential revisions:

The reviewers agreed that this is a valuable and interesting study (see reviews below). They suggested additional analyses that would strengthen the main conclusion of the study, because the clustering methods are not conclusive about the presence of distinct subpopulations. The thought is that any additional evidence in support of the central claims would be helpful.

1) The authors should consider separation metrics that have been used in previous studies (the silhouette score and the adjusted rand index) and compare the optimal number of clusters found with these metrics with their analysis using the gap statistics. This would give better insight into the parameters controlling the complexity of the responses at the level of populations. See comment from Rev 2.

We investigated the silhouette score and adjusted rand index for our features space representation of lOFC responses. Both methods suggested that 2 clusters were present in our dataset. Using additional analyses we demonstrated that neither method is well-suited to make a principled choice of cluster size for our lOFC response. The adjusted rand index, a measure of reproducibility of a clustering result, demonstrated that a large range of cluster sizes (2-8) could be robustly identified in our data, including our primary result of 5 clusters. Therefore, the results from this metric were not definitive about the number of clusters. The silhouette score did not have a clear peak for a specific number of clusters, and instead exhibited a monotonic decay for larger cluster numbers. The silhouette score is known to be inaccurate in certain data regimes (see Garcia-Dias et al., 2018 and 2020), and should be utilized only when the silhouette score results are definitive. The silhouette score is designed to locate clustered data that is both tightly packed together within a cluster, but also well separated and distanced from neighboring clusters. We hypothesized that this penalty for “crowded” clusters may be responsible for our inconclusive silhouette score result, and performed a study on ground-truth data with varying cluster spacing. We found that the silhouette score consistently underestimated the number of clusters in this study in the regime of lOFC responses, and reproduced the same decaying silhouette score values as in our data. We also found that the gap statistic underestimated the number of clusters in ground truth data, but did so to a much lesser degree. This conservative estimation of cluster numbers may be responsible for the discrepancy between our result of 5 clusters, and larger cluster numbers from other groups (*e.g*., Hirokawa et al. 2019).

The changes to the manuscript were the following:

– Reporting of the silhouette score and adjusted rand index is given in a Figure 2-figure supplement 5.

– Results of a ground-truth synthetic data study of the silhouette score is provided in Figure 2-figure supplement 6.

– A comparison of these methods, as well as a justification for utilizing the gap statistic over the other methods, is provided in the Results section.

– A description of how we calculated the silhouette score and ARI is provided in the Methods section.

– A description of the ground-truth study formulation is provided in the Methods section.

2) It would also be interesting if the authors compared the properties of neurons in cluster 3 to those of striatum-projecting neurons (and their associated cluster) found in a previous study (Hirokawa et al., 2019). Potentially, this could show that the clustering methods presented here can robustly identify similar populations of neurons across behavioral tasks, and would also provide a potential mechanistic basis for the learning effects mediated by OFC. See comment from Rev 2.

We investigated if particular clusters in our data had similar encoding properties to the striatum-projecting neurons that were identified in (Hirokawa et al., 2019). In that work, those neurons encoded the reward outcome following reward delivery, with larger responses for unrewarded trials, and also persistently encoded negative integrated value during the inter-trial interval until the start of the next trial. We evaluated the cluster-averaged responses for different reward volumes at reward delivery and trial start, and found that while several clusters encoded reward outcome after reward delivery, only cluster 3 also encoded the magnitude of reward volume during the inter-trial interval. Moreover, cluster 3 neurons exhibited qualitatively similar encoding to the corticostriatal cells from Hirokawa et al., in which they exhibited smaller responses for larger rewards, and the largest responses following unrewarded trials. We believe that this cluster may correspond to striatum-projecting neurons. We discuss these implications, including the possibility of a neural substrate of sequential learning effects, further in the Discussion section.

The changes to the manuscript were the following:

– We included a new section in the Results section that describes the corticostriatal projection neurons and their encoding properties from Hirokawa et al.*, 2019,* and added Figure 7 to the Results section, which compares our cluster-averaged responses for different reward volumes.

– We discuss the implications of cluster 3 being a potential set of striatum-projecting neurons in the Discussion section.

– We added this primary result to the abstract of the manuscript.

3) If data are available/appropriate, determine whether neurons have narrow or broad spikes, thus providing another potential criterion for characterizing the clusters. See comment from Rev 3.

We have included additional analysis on the waveform. We adopted an analysis from Bruno and Simons (J. Neuroscience, 2002) in which we compared the widths of the action potential (AP) and the after-hyperpolarization (AHP) activity. Similar to that work, we found two clusters of neurons when looking at AP and AHP: One cluster of neurons contained shorter AP and AHP activity, while a second cluster contained slower AP and AHP activity. When using AP and AHP widths as potential criterion for further characterizing our 5 clusters from the main text of the manuscript, we found no relationship between slow or fast single units and our 5 clusters. Specifically, we found that the distribution of the two cell types was similar across clusters.

The changes to the manuscript were the following:

– We added Figure 4-figure supplement 1, which shows the distribution of putative regular and fast-spiking cells across clusters, as well as details of the waveform analysis.

– We added a description of how we performed the analysis in the Methods Section.

– We added a brief section to the Results section.

4) In Figure 2C it seems that clusters largely differ by their late responses at the end of the trials. Does a cluster analysis based on the late parts of the PSTHs lead to similar results to those found? See comment from Rev 3.

We performed clustering based on PSTHs aligned to when the animal leaves the center port to make a choice. Assessment of cluster size using the Gap statistic yielded a similar number of clusters, K=6. The partitioning of units based on choice-aligned PSTHs was very similar to that based on PSTHs aligned to the start of the trial. Furthermore, it revealed a noteworthy, finer-scale structure to the reward history encoding seen late in the trial: The additional cluster from this analysis partitioned reward history encoding to just before choice (cluster 3), and precisely at choice (cluster 5). Given that this was the only major distinction between encoding of task attributes between the two clustering approaches, we have kept our original analyses, using responses aligned to trial start, in the main text, and have added the results of this late-in-trial clustering to the Supplemental Information.

The changes to the manuscript were the following:

– We added the results of this new clustering approach in Figure 5-figure supplement 2, and mentioned them in the Results section.

5) The endorsement of adaptive value coding as something that OFC is dedicated to is perhaps a bit too optimistic, considering that only 15% of neurons demonstrated it (see comment from Rev 1). The authors should consider a more balanced discussion of this point.

The changes to the manuscript were the following:

– We have relaxed our interpretation of adaptive value coding being dedicated to OFC in the Discussion section. We acknowledge that 15% is a modest fraction of neurons, and emphasize that adaptive value coding is probably not specific to OFC, but occurs broadly in brain areas representing subjective value.

6) The authors mention that the neurons in cluster 3 might support the integration of reward signals, but it is largely unclear why, especially from a computational point of view. Why do history and current trial reward signals ought to be integrated in this task? Spelling this out would be useful.

Given the new result that cluster 3 neurons exhibit qualitatively similar responses to striatum-projection neurons (Hirokawa et al., 2019), we have included a discussion of how these representations of reward history and current reward outcome may affect trial-by-trial learning. Specifically, we discuss the implications of these coincident signals in the context of reinforcement learning accounts of the basal ganglia, in which corticostriatal projection neurons may be representing information about the animal’s state, and corticostriatal synapses represent that value of performing particular actions in that state (Q-values). Coincident activation of cortical inputs and striatal spiking would allow synapses to be tagged for plasticity in the presence of dopamine, thereby modulating state-action values with experience. We speculate that if reward history and current trial reward signals are coincidently represented, then contextual inputs to the striatum that reflect the animal's “state,” and that could be plastically modified in the presence of dopamine, would include the conjunction of previous and current rewards (i.e., I was rewarded on the previous trial, and then rewarded again. I am on a winning streak).

While there is no need for trial-by-trial learning in this task, as reward contingencies are independent and explicitly cued on each trial, the evolutionary importance of learning about changes in dynamic environments may introduce these sub-optimal, sequential biases.

An alternative possibility, which we also discuss, is that the representations of reward history at the time of choice influence ongoing neural dynamics -in lOFC or downstream- that support the current choice, as in Mochol et al., 2021.

The changes to the manuscript were the following:

– We added additional text to the Discussion section where we describe representations of reward history.
